# Report and follow-up on two new patients with congenital mesoblastic nephroma

**DOI:** 10.1186/s13052-023-01523-7

**Published:** 2023-09-19

**Authors:** Gregorio Serra, Marcello Cimador, Mario Giuffrè, Vincenzo Insinga, Claudio Montante, Marco Pensabene, Ettore Piro, Sergio Salerno, Ingrid Anne Mandy Schierz, Giovanni Corsello

**Affiliations:** Department of Health Promotion, Mother and Child Care, Internal Medicine and Medical Specialties “G. D’Alessandro”, University of Palermo, Palermo, Italy

**Keywords:** CMN, Kidney tumors, Neonate, Paraneoplastic syndrome, Intestinal occlusion, Case report

## Abstract

**Background:**

Tumors are rare in neonatal age. Congenital mesoblastic nephroma (CMN) is a usually benign renal tumor observed at birth, or in the first months of life. It may also be identified prenatally and associated with polyhydramnios leading to preterm delivery. Effective treatment is surgical in most cases, consisting in total nephrectomy. In literature, very few studies report on the neonatal management of such a rare disease, and even less are those describing its uncommon complications.

**Cases presentation:**

We report on two single-center newborns affected with CMN. The first patient is a preterm female baby, born at 30^+ 1^ weeks of gestation (WG) due to premature labor, with prenatal (25 WG) identification of an intra-abdominal fetal mass associated with polyhydramnios. Once obtained the clinical stability, weight gain, instrumental (computed tomography, CT, showing a 4.8 × 3.3 cm left renal neoformation) and histological/molecular characterization of the lesion (renal needle biopsy picture of classic CMN with *ETV6-NTRK3* translocation), a left nephrectomy was performed at 5 weeks of chronological age. The following clinical course was complicated by intestinal obstruction due to bowel adherences formation, then by an enterocutaneous fistula, requiring multiple surgical approaches including transitory ileo- and colostomy, before the conclusive anastomoses intervention. The second patient is a 17-day-old male term baby, coming to our observation due to postnatal evidence of palpable left abdominal mass (soon defined through CT, showing a 7.5 × 6.5 cm neoformation in the left renal lodge), feeding difficulties and poor weight gain. An intravenous diuretic treatment was needed due to the developed hypertension and hypercalcemia, which regressed after the nephrectomy (histological diagnosis of cellular CMN with *ETV6-NTRK3* fusion) performed at day 26. In neither case was chemotherapy added. Both patients have been included in multidisciplinary follow-up, they presently show regular growth and neuromotor development, normal renal function and no local/systemic recurrences or other gastrointestinal/urinary disorders.

**Conclusions:**

The finding of a fetal abdominal mass should prompt suspicion of CMN, especially if it is associated with polyhydramnios; it should also alert obstetricians and neonatologists to the risk of preterm delivery. Although being a usually benign condition, CMN may be associated with neonatal systemic-metabolic or postoperative complications. High-level surgical expertise, careful neonatological intensive care and histopathological/cytogenetic-molecular definition are the cornerstones for the optimal management of patients. This should also include an individualized follow-up, oriented to the early detection of any possible recurrences or associated anomalies and to a better quality of life of children and their families.

## Background

Kidney tumors are rare in developmental age, accounting for 6–7% of all children’s neoplasms. Nephroblastoma or Wilms’ tumor (WT) is the most commonly observed throughout the whole pediatric age group, representing the 90% of all cases [1], while congenital mesoblastic nephroma (CMN) is the most frequent within the first 5 months of life, accounting for 54% of the renal tumors in neonatal age [1, 2]. Other forms, which include the lethal rhabdoid tumor (RTK), are rarely found [1, 3]. No specific ultrasonographic features allow CMN differentiation from other renal neoformations. Actually, the most suggestive aspect is its appearance in the fetal period, which is less frequent in other renal neoplasms [4–6]. Some additional information could be provided by fetal Magnetic Resonance Imaging (MRI), which however is not part of the routine diagnostic work-up [4, 7, 8]. After birth, other exams like tumor markers and needle biopsy are available and may be used to reach the diagnosis [4, 9–11]. However, histopathological evaluation by surgery is the diagnostic gold standard [1]. CMN has excellent prognosis and nephrectomy is usually sufficient for effective treatment [1, 12].

Herein, we report on two newborns affected with CMN, both observed at the Mother and Child Department of the University Hospital of Palermo. Our study underlines the diagnostic critical issues faced to promptly carry out second level imaging investigations (computed tomography, CT), as well as invasive exams (needle biopsy) before surgery. It also highlights the complex treatment of such a rare disease: both patients underwent surgery at variable times after birth, based on their different gestational age, weight and overall clinical condition, they showed systemic (metabolic alterations, arterial hypertension) or abdominal postoperative complications which required additional medical and surgical interventions.

## Cases presentation

### Patient 1

**Fig. 1:**
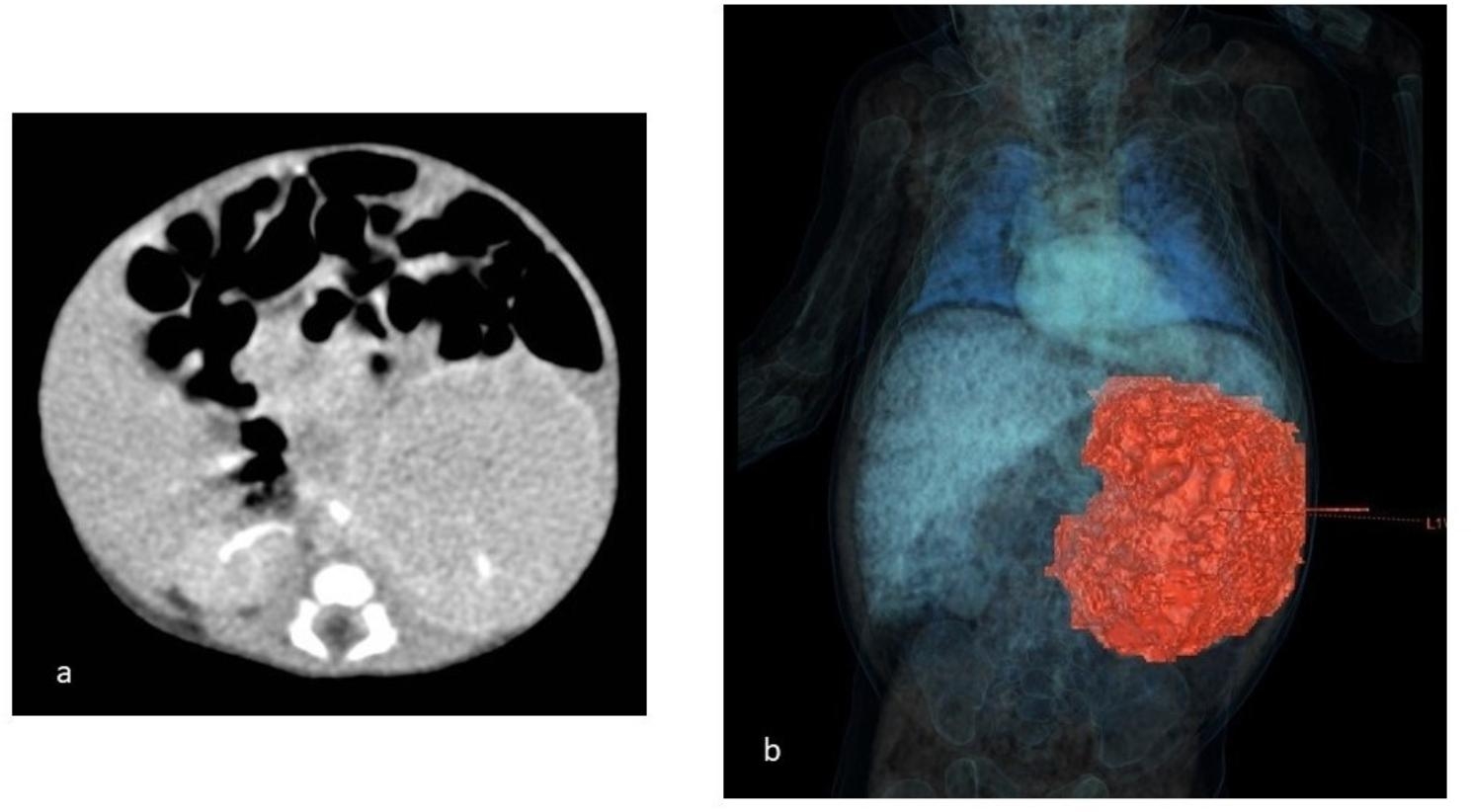
Patient 1. **a** Abdominal computed tomography shows the left renal mass (corresponding to a volume of 76 mL), and defines its relationships with the surrounding organs and structures. **b** Three-dimensional CT reconstruction

**Fig. 2:**
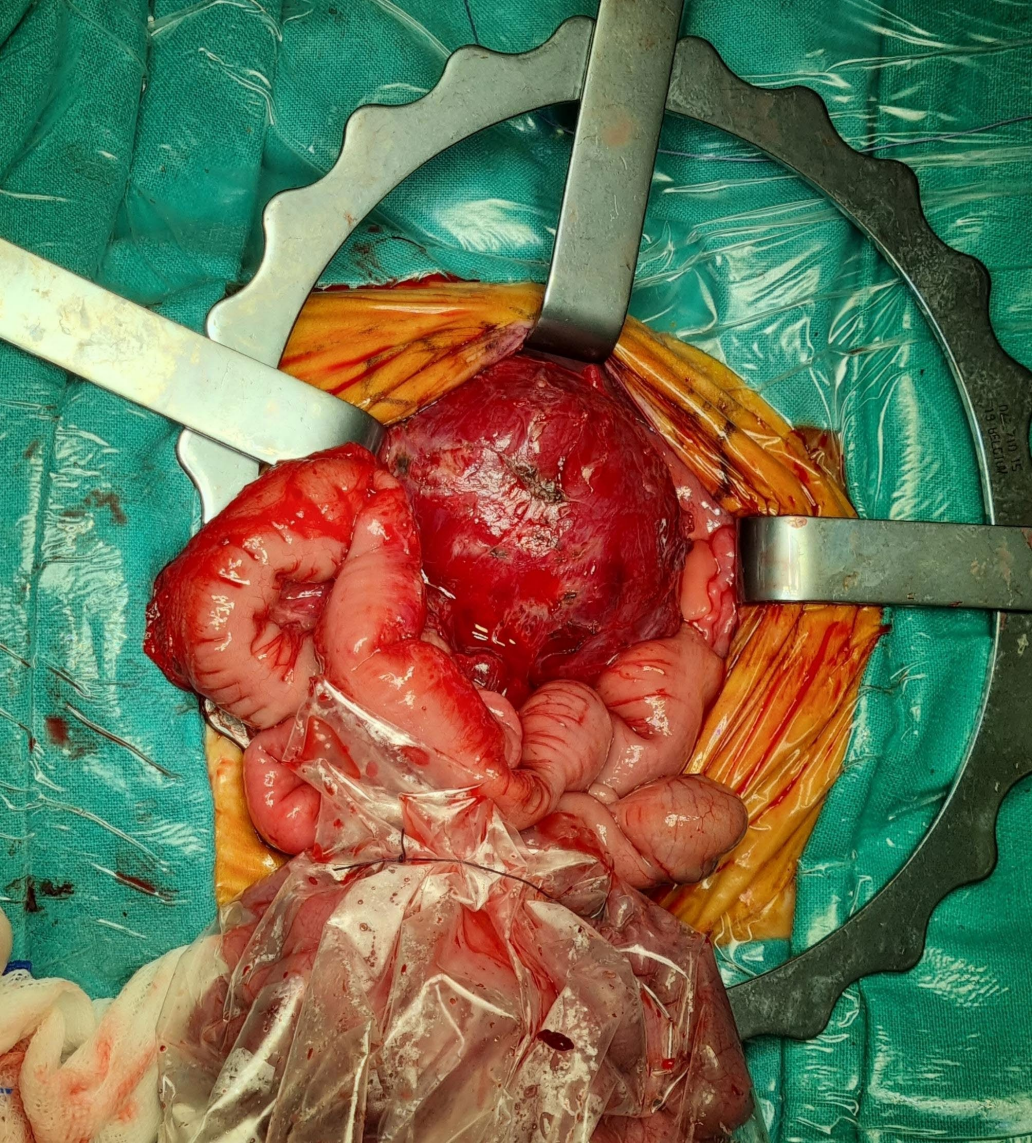
Patient 1. Intraoperative view of the left nephrectomy

A female preterm infant was born by spontaneous vaginal delivery at 30^+ 1^ weeks of gestation (WG), due to premature labor. An intra-abdominal fetal mass with probable origin from the left kidney, along with polyhydramnios, was detected by prenatal ultrasound (US) investigations at 25 WG; the following evaluation at 27 WG showed an increased size and echogenicity of the mass within the left kidney, leading to steroid prophylaxis (two doses of intramuscular betamethasone, 12 mg 24 h apart) in the mother two weeks later, due to the increased risk of preterm delivery. At birth, anthropometric measures were as follows: weight 1,450 g (75th centile, + 0.69 standard deviations, SD), length 41 cm (86th centile, + 1.1 SD), occipitofrontal circumference (OFC) 28.3 cm (77th centile, + 0.75 SD). Postnatally, the newborn manifested mild respiratory distress, which required non-invasive ventilatory support for the first 72 h of life. Chest X-ray examination did not show either elevation of the diaphragm/thoracic compression or signs of pulmonary hypoplasia. Meanwhile, for the first three days, a total parenteral nutrition was given, after which enteral feeding was begun with good tolerance and spontaneous stool emission. Physical examination showed a palpable mass in the left side of the abdomen and no other abnormalities (no hemihypertrophy or other dysmorphic features), which did not lead, then, to perform any genetic investigations (methylation test or next generation sequencing analysis of the genes associated with overgrowth syndromes) [13, 14]. Abdominal US localized the lesion within the left renal lodge. It measured 4.8 × 3.3 cm, and showed inhomogeneous echogenicity and intralesional vascularity without infiltration of the vascular pedicle. The right kidney appeared normal, and no involvement of other organs was observed. Computed Tomography (CT) confirmed the size of the renal mass (corresponding to a volume of 76 mL), it also identified inhomogeneous and peripheral enhancement. Its relationships with colon, splenic vein and pancreas were better defined, calcifications as well as cystic areas were ruled out (Fig. 1a/b). Heart US showed normal findings. Complete blood count, renal function tests, serum alpha-fetoprotein (AFP), beta-human chorionic gonadotropin (beta-HCG) and neuron-specific enolase (NSE) assays, along with urinary catecholamines showed normal results. During the first 2 weeks of life, the clinical course was marked by hypercalcemia (13.86 mg/dL, normal values [n.v.] for preterm infants 7–11 mg/dL) and arterial hypertension (mean blood pressure value 69 mmHg, 99th centile) [15], which were treated with intravenous furosemide, due to its diuretic effect based on increased urinary calcium excretion. At age 3 weeks (33 weeks of corrected age), a left renal needle biopsy was performed. Histopathological evaluation showed mesenchymal proliferation of monomorphic, oval, spindle-shaped cells arranged in intertwined bundles, consistent with classic CMN diagnosis. The *ETV6-NTRK3* genes translocation was found. Aged 5 weeks (35 weeks of corrected age) the patient underwent left nephrectomy (Fig. 2), and histological examination confirmed the classic CMN diagnosis. No chemotherapy was started. The early postoperative course was regular, with rapid tolerance of enteral feeding and normal stool emission. However, 26 days after nephrectomy, a clinical picture of acute abdomen appeared including vomiting, failure to pass stool and abdominal protrusion. X-ray evidenced distension of the ileal and colic loops, as well as hydro-aerial levels. Therefore, a laparotomy was performed, disclosing tight bands around the descending-sigmoid colon which required a transitory colostomy. The biopsied intestinal and lymph node tissues identified no lesions referring to CMN. The subsequent clinical evolution was marked by the occurrence of an enterocutaneous fistula due to ileal perforation, which needed surgical closure, packaging of an ileostomy, as well as antibiotic treatment. The following clinical course occurred without complications: enteral feeding with an amino acid-based formula was well tolerated [16]; adequate weight gain was observed, while the stool emission had been allowed through the colostomy. Thus, at age 5 months and 27 days (3 months and 17 days of corrected age), ileo-ileal and colo-colic anastomoses surgery was performed. The patient was discharged at 6 months and 15 days (4 months and 6 days of corrected age) and included in a multidisciplinary follow-up. Now aged 20 months (18 months of corrected age), she shows regular growth (weight 10,220 g, 48th centile, -0.04 SD; length 82 cm, 64th centile, + 0.36 SD; OFC 44.5 cm, 10th centile, -1.28 SD) – according to World Health Organization growth chart for neonatal and infant close monitoring [17] – and neuromotor development. Currently, the renal function tests are normal, and she has no local/systemic recurrences or other gastrointestinal disorders.

### Patient 2

**Fig. 3:**
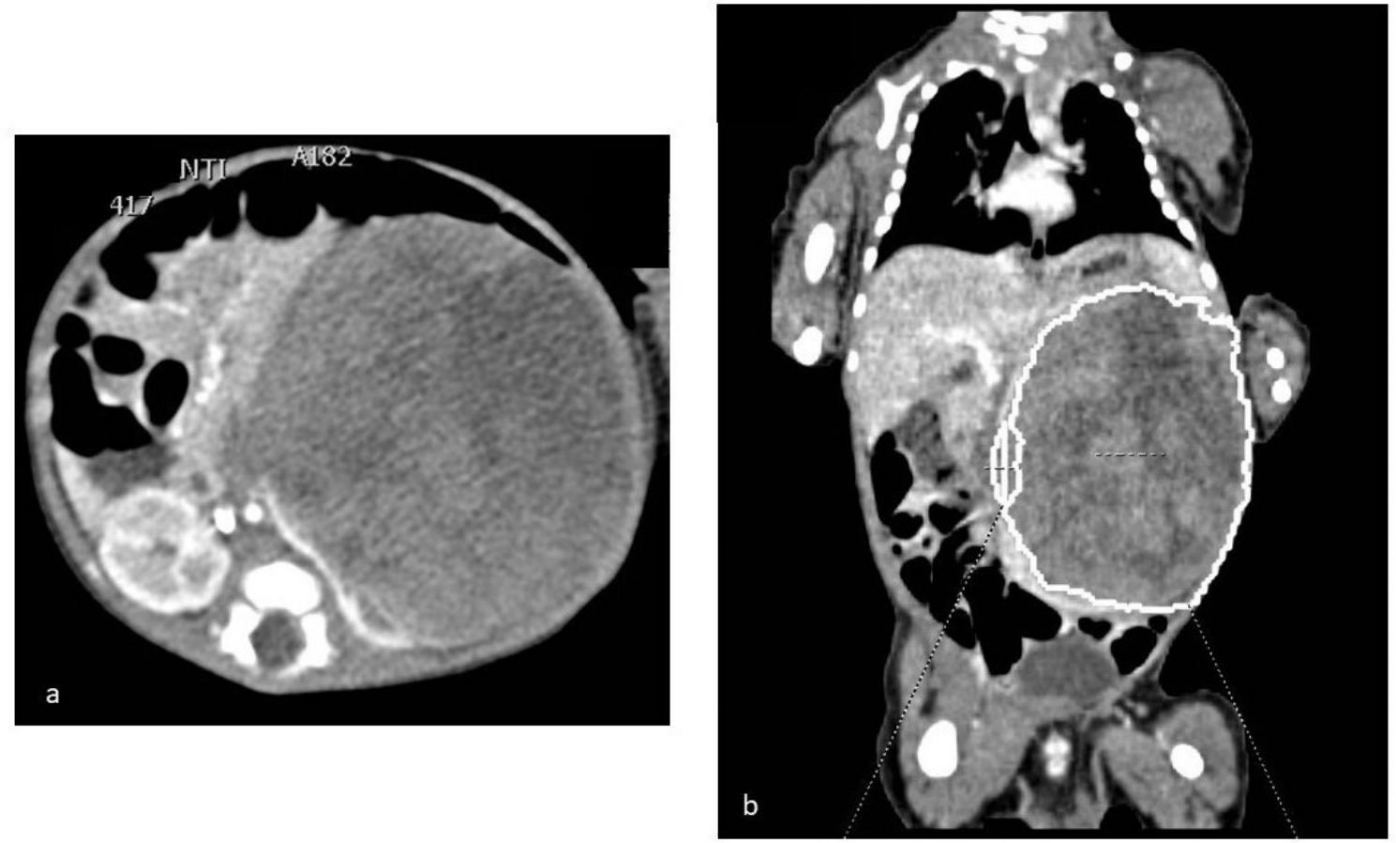
Patient 2. Abdominal (**a**) and chest (**b**) CT shows a mass in the left renal lodge, without lymph node enlargement/infiltration or secondary pleuropulmonary lesions

A 17-day-old term male newborn was admitted to our Department due to palpable left abdominal mass, feeding difficulties and poor weight gain. Pregnancy and perinatal history were unremarkable. At admission, physical examination showed severe global growth restriction, including weight (2,480 g; <0.4th centile, -2.9 SD), length (47 cm; <0.4th centile, -2.9 SD) and OFC (33 cm; <0.4th centile, -2.6 SD) [17], prominent abdomen with visible venous reticulum and a palpable mass within the left abdominal side. No other abnormalities were observed. Abdominal US documented a solid mass in the left kidney measuring 7.5 × 6.5 cm, with hypoechoic areas of necrosis without infiltration of the vascular pedicle. Abdominal CT better defined the neoformation, which was located in the left renal lodge and characterized by inhomogeneous enhancement and absence of lymph node enlargement or infiltration **(**Fig. 3a**)**. Chest CT excluded secondary pleuropulmonary lesions (Fig. 3b). Heart US showed normal findings. Blood tests detected leukocytosis with lymphocytosis (total white blood cells 24,930/mL; lymphocytes 15,170/mL), increased levels of lactic dehydrogenase (374 U/L, n.v. 50–250), hypercalcemia (14.36 mg/dL; n.v. for term babies 8–11 mg/dL), in addition to mild elevation of serum phosphorus and magnesium levels (5.39 mg/dL and 2.45 mg/dL, n.v. 2.5–4.5 and 1.5–2.2, respectively). Therefore, intravenous administration of furosemide was started. Serum AFP, beta-HCG and NSE levels, as well as urinary catecholamines, were within normal range. During the first week of the hospital stay, our patient presented severe arterial hypertension (mean blood pressure value 88 mmHg, > 99th centile) [15], associated with secondary hyperaldosteronism due to hyperreninemia (280 ng/L; n.v. 2.99–35.64 ng/L). However, no abnormalities of plasmatic sodium, potassium and acid-base equilibrium (ABE) were detected. In addition, urine analysis excluded microhematuria. At 26 days of life, the patient underwent left nephrectomy. Subsequent histological examination disclosed a neoformation characterized by oval and spindle-shaped cells arranged in bundles, with necrosis and mitotic activity of 10/10 per high-power field; molecular analysis revealed the *ETV6-NTRK3* fusion. Therefore, based on such findings, the cellular CMN diagnosis was made, and no chemotherapy was added. The postoperative evolution was characterized by an immediate rebound hypocalcemia and hypotension which required intravenous calcium correction, inotropes and eventually, blood transfusions and albumin administration. He was discharged 16 days after nephrectomy, showing regular feeding and adequate growth. Aged 4 years, our patient is presently included in a multidisciplinary follow-up. He shows normal anthropometric parameters and neuromotor development, kidney function profile, no recurrences nor other abnormalities.

## Discussion and conclusions

CMN is the most frequently diagnosed renal tumor in the first 5 months of life, and it may also be evidenced prenatally [1]. However, it is rarely detected by mid-pregnancy US (20 WG) [7, 18], while more commonly it may be indirectly revealed through the co-occurrence of polyhydramnios, commonly observed during the third trimester [4]. In the present patients, polyhydramnios was found only in the first case, referring to the fetal left renal mass. In the second case, no antenatal diagnosis was made; the first signs of disease were an asymptomatic palpable abdominal mass, along with poor feeding and growth. Polyhydramnios can result in preterm labor and delivery, as occurred in our first case, contributing to a more complex management of patients due to the neonatological issues associated with prematurity.

Urinary catecholamine assay and serum NSE resulted normal in both our patients, they are essential since neuroblastoma represents the most frequent extracerebral solid tumor in newborns and children. Therefore, it must be first included in the differential diagnosis of abdominal masses. Other oncological diseases, from which CMN should be distinguished in the neonatal period, are different kidney tumors (mainly Wilms) and germ cell ones (GCTs) to which teratomas, yolk sac tumors and choriocarcinomas belong. Indeed, other biochemical markers including serum AFP and beta-HCG should be evaluated [19]; in both reported cases they showed normal results.

Histologically, CMN may be classified into three subtypes: the cellular type is the most common, followed by the classic and mixed ones. The former is the biologically most aggressive and is associated with higher risk of recurrences and metastases [20, 21]. In both our cases, histology was performed to define the diagnosis. From a genetic point of view, 70% of CMNs are characterized by (12;15)(p13;q25) translocation, which causes fusion of the ETS variant 6 (*ETV6*) and Neurotrophic Receptor Tyrosine Kinase 3 (*NTRK3*) genes; the oncogene resulting from this translocation, encodes for an oncogenic chimeric protein that would be responsible for tumorigenesis, through dysregulation of NTKR3 signal transduction pathways and for chemosensitivity of cancer cells [1, 20, 22–24]. The *ETV6-NTRK3* gene fusion is also a key marker which allows to distinguish CMN from other renal tumors, as well as the cellular subtype (it is mainly detected in this form [25, 26]) from the other two variants. Such translocation is moreover a prognostic factor associated with higher relapse free-survival (RFS) compared with translocation-negative forms (5-year RFS 100% vs. 73%, respectively) [26]; therefore, genetic study may stratify patients with cellular CMNs, identifying those with higher risk of recurrence, for which additional chemotherapy treatment might be considered [27]. This rearrangement was identified in both our patients, who did not perform any chemotherapy treatment, indeed. It is reported a five-year event-free survival (EFS) rate of 94% and an overall survival (OS) rate of 96% in all CMN cases, with lower expectations for the cellular subtype (85% and 90% of RFS and OS, respectively [6]). Poorer prognoses are associated with chemotherapy toxicity and postoperative complications. Nephrectomy is the treatment of choice; chemotherapy and radiotherapy are rarely required. Critical issues may rise for newborn patients, especially preterm, who require multispecialty co-management of neonatologists, anatomopathologists, pediatric surgeons, oncologists and nephrologists. Actually, in the first patient the clinical instability due to prematurity and to the low birth weight delayed nephrectomy; the classic CMN diagnosis has been provided through needle biopsy. Conversely, in patient 2 nephrectomy has been soon performed, and histological examination was made after surgical removal of the lesion. In patient 1, the postoperative evolution was complicated by acute abdomen due to bowel occlusion as well as by an enterocutaneous fistula, which required further surgical approaches including the packaging of ileo and colostomy. A case of ileocolic intussusception is reported in a 6-month-old infant with CMN, who underwent left nephrectomy. Other CMN patients with intestinal occlusion secondary to nephrectomy or with different complications are poorly reported in literature (and synthetized in Table 1), likely due to both the rarity of disease and the low incidence of postoperative issues, especially in otherwise healthy infants [28]. Also, associations with a prevalent side localization have been previously described, but data are conflicting (Table 1).

**Table 1:** Overview of the previous studies on CMN associated with neonatal/infant complications

References	Diagnosis	Side	Prenatal finding of abdominal mass	Polyhydramnios	Preterm labor (gestational age)	Age at diagnosis	Complications
Kato H, 2022 [7]	CMN (not specified)	Right	+	+	+ 36 WG	preterm newborn (36 WG)	Abdominal compartment syndrome, respiratory distress
Soyaltın E, 2018 [29]	Cellular CMN	Left	+	-	+ 32 WG	preterm newborn (32 WG)	Refractory hypertension
Kim JS, 2017 [28]	CMN (not specified)	Left	-	not specified	not specified	infant (6-month-old)	Persistent hypertension Right lower quadrant ileocolic intussusception
Kamaraj S, 2016 [30]	Cellular CMN	Right	-	-	-	term newborn (2-day-old)	Hematuria
Daskas N, 2002 [31]	Mixed CMN	Right	+	+	+ 33 WG	preterm newborn (33 WG)	Hypercalcemia
Fung TY, 1995 [32]	CMN (not specified)	Left	+	+	-	term newborn (40 WG)	Hypercalcemia, polyuria and polyhydramnios
Zach TL, 1991 [33]	Mixed CMN	Right	-	+	-	term newborn (38 WG)	Hemorrhagic shock and disseminated intravascular coagulation

+ = present; - = absent; WG = weeks of gestation

In patient 2, hypercalcemia and arterial hypertension were observed. In literature, both complications are reported associated with CMN, due to secretion of prostaglandins, parathyroid hormone, parathyroid-related and glucagon-like peptides, as well as renin released by tumor cells. Both conditions required medical therapy with furosemide and were promptly resolved after tumor removal, highlighting that their pathogenesis is related with factors secreted by tumor cells (paraneoplastic syndromes) [21, 29, 34]. The underlying pathophysiological mechanism of hypertension is the secondary hyperaldosteronism, due to increased plasma renin activity (PRA); therefore, serum electrolytes and acid-base equilibrium (ABE) should be monitored, owing to the higher risk of hypokalemia and metabolic alkalosis. Furosemide, which was used in both our patients, is considered as the first choice drug in the treatment of hypercalcemia and hypertension, due to its effect in promoting urinary calcium excretion; in case of refractory hypertension, co-administration of calcium channel blockers (e.g., nifedipine and amlodipine) may be considered more appropriate [29].

The present study highlights that, although CMN is usually a benign condition, it may be associated with complications in the neonatal period; they may arise post-surgically or be systemic-metabolic and require different approaches, also in relation with the potential co-occurrence of prematurity/low birth weight and of their related morbidities. Thus, an early (even prenatally) multidisciplinary management, as for other congenital diseases [35–39], is essential to offer the patients the most adequate treatment. Actually, the finding of a fetal abdominal mass should prompt suspicion of CMN, especially if it is associated with polyhydramnios, and it must alert obstetricians and neonatologists also to the risk of preterm delivery. After birth, multispecialty co-management of newborn patients, which includes the integration of high-level surgical expertise with careful neonatological intensive care [40–45], is necessary to limit and/or prevent complications. Finally, the precise definition of the histopathological and cytogenetic-molecular profiles [46–54] is indispensable to plan an individualized follow-up, oriented to early detection of any possible recurrences or associated anomalies and to better quality of life for children and their families [55–57].

## Data Availability

The datasets used and analyzed during the current study are available from the corresponding author on reasonable request.

## List of Abbreviations

ABE: Acid-base equilibrium
AFP: Alpha-fetoprotein
Beta-HCG: Beta-human chorionic gonadotropin
CMN: Congenital mesoblastic nephroma
CT: Computed tomography
*ETV6*: ETS variant 6
GCTs: Germ cell tumors
NSE: Neuron-specific enolase
*NTRK3*: Neurotrophic Receptor Tyrosine Kinase 3
PRA: Plasma renin activity
MRI: Magnetic resonance imaging
US: Ultrasonography
WG: Weeks of gestation
WT: Wilms’ tumor
